# The Hemoglobin, Albumin, Lymphocyte, and Platelet (HALP) Score Is Associated With Poor Outcome of Acute Ischemic Stroke

**DOI:** 10.3389/fneur.2020.610318

**Published:** 2021-01-12

**Authors:** Mengke Tian, Youfeng Li, Xiao Wang, Xuan Tian, Lu-lu Pei, Xin Wang, Luyang Zhang, Wenxian Sun, Jun Wu, Shilei Sun, Mingming Ning, Ferdinando Buonanno, Yuming Xu, Bo Song

**Affiliations:** ^1^Department of Neurology, the First Affiliated Hospital of Zhengzhou University, Zhengzhou, China; ^2^Henan Key Laboratory of Cerebrovascular Diseases, Zhengzhou, China; ^3^Massachusetts General Hospital and Harvard Medical School, Clinical Proteomics Research Center and Cardio-Neurology, Boston, MA, United States

**Keywords:** acute ischemic stroke, cohort study, HALP score, outcome, risk factors

## Abstract

**Background:** The combined index of hemoglobin, albumin, lymphocyte, and platelet (HALP) is considered a novel score to reflect systemic inflammation and nutritional status. This study aimed to investigate the association between HALP score and poor outcome in patients with acute ischemic stroke (AIS).

**Methods:** Consecutive AIS patients within 24 h after onset were prospectively enrolled. Poor outcome was a combination of a new stroke event (ischemic and hemorrhagic) and all-cause death within 90 days and 1 year. The association between HALP score and poor outcome was analyzed using Cox proportional hazards.

**Results:** A total of 1,337 patients were included. Overall, 60 (4.5%) and 118 (8.8%) patients experienced poor outcome within 90 days and 1 year, respectively. Patients in the highest tertile of HALP score had a lower risk of poor outcome within 90 days and 1 year (hazard ratio: 0.25 and 0.42; 95% confidence intervals: 0.11–0.57 and 0.25–0.69, *P* for trend <0.01 for all) compared with those in the lowest tertile after adjusting relevant confounding factors. Adding HALP score to the conventional risk factors improved prediction of poor outcome in patients with AIS within 90 days and 1 year (net reclassification index, 48.38 and 28.95%; integrated discrimination improvement, 1.51 and 1.51%; *P* < 0.05 for all).

**Conclusions:** Increased HALP score was associated with a decreased risk of recurrent stroke and death within 90 days and 1 year after stroke onset, suggesting that HALP score may serve as a powerful indicator for AIS.

## Introduction

Stroke is a leading cause of death and long-term disability worldwide, with ischemic stroke as the most common subtype (1). Exploring new predictors of stroke prognosis may help in early identification of high-risk patients with poor outcome and contribute to more effective secondary prevention. Inflammation, abnormal blood coagulation, and poor nutritional status are associated with poor prognosis of acute ischemic stroke (AIS) (2–4). Lymphocytes have key regulatory functions in post-stroke inflammation (5). Platelet hyperactivity increases the risk of thromboembolism and atherosclerotic lesions and may lead to abnormal thrombosis, which exacerbates inflammation response (6). Anemia and hypoalbuminemia are manifestations of poor nutritional status and have been identified as risk factors for cerebrovascular events that may lead to poor prognosis in patients with AIS (7, 8). In this study, we explored the association between the combination of these four common indicators and the prognosis of AIS.

The hemoglobin, albumin, lymphocyte, and platelet (HALP) score is considered to be an easily calculated marker of systemic inflammation and nutritional status (9, 10) and has been found as a significant prognostic factor in patients with multiple tumors (11–13). However, it is unknown whether HALP score is associated with the poor outcome of AIS. Therefore, our study aimed to investigate the predictive value of HALP score in a prospective cohort of patients with AIS.

## Methods

### Study Design and Population

The study was a prospective consecutive hospital-based cohort study. We enrolled patients with AIS within 24 h of onset from the Ischemic Cerebrovascular Disease Database of the First Affiliated Hospital of Zhengzhou University from January 2015 to June 2018, which has been published previously (14, 15). The diagnosis of AIS was based on the criteria of the World Health Organization (WHO) (16). The database was approved by the Ethics Committee, and informed consent forms were obtained from all patients or their relatives.

The exclusion criteria were as follows: (i) patients with active or chronic inflammatory disease; (ii) neoplastic hematologic disorders or using immunosuppressant drugs; (iii) major trauma or surgery within 3 months; (iv) patients without blood cell count data; and (v) severe liver and kidney dysfunction.

### Clinical Assessment

Baseline clinical information was obtained from case report forms at admission, including demographic characteristics, medical histories, imaging features, and medication use. Clinical factors, including baseline stroke severity, the use of intravenous thrombolysis therapy, and stroke subtype, were also evaluated. The severity of AIS was assessed according to the National Institutes of Health Stroke Scale (NIHSS) at admission. Stroke subtypes were classified according to the Trial of Org 10172 in the Acute Stroke Treatment classification.

### Laboratory Assay

Laboratory tests were obtained within 24 h of admission after stroke onset, including the serum albumin, hemoglobin, lymphocyte, and platelet levels. The blood counts were analyzed using an autoanalyzer (Beckman Coulter Hematology Analyzer LH750, USA). All the serum biochemical parameters were assayed using an automatic biochemical analyzer (Roche COBAS 8000 automatic biochemical analyzer). The HALP score was calculated according to the following formula: hemoglobin (g/L) × albumin (g/L) × lymphocytes (/L)/platelets (/L).

### Outcome Assessments

The poor outcome was a combination of a new stroke event (ischemic and hemorrhagic) and all-cause death within 90 days and 1 year. Patients enrolled were followed up by face-to-face or telephone interviews at 90 days and 1 year after stroke onset.

### Statistical Analyses

Continuous variables with normal distribution were expressed as mean ± standard deviation (SD), and skewed distribution were expressed as medians with interquartile ranges (IQRs). Categorical variables were expressed as frequencies (*n*) and percentages (%). Patients were classified into three groups based on the HALP score tertiles. Tests for linear trend of baseline characteristics across HALP score tertiles were performed using ANOVA for continuous variables and χ^2^ trend analysis for categorical variables. The cumulative incidence risks of poor outcome across baseline HALP score were calculated with Kaplan–Meier curves and compared by log-rank test. Multivariate Cox proportional hazards models were used to assess the risk of poor outcome with HALP score tertiles. Unadjusted and adjusted hazard ratios (HRs) and their 95% confidence intervals (CIs) were calculated for the two higher HALP score groups (with the lowest group serving as a reference) and for a 1-SD increment of HALP score. Optimal HALP score cutoff values were obtained using receiver operating characteristic (ROC) curve analysis. C statistics, net reclassification improvement (NRI), and integrated discrimination improvement (IDI) were used to evaluate the incremental predictive ability of HALP score beyond the conventional model. Some conventional risk factors were selected as potential covariates for poor outcome of ischemic stroke based on prior knowledge. In addition, we used restricted cubic splines to evaluate the shape of the association between HALP score and outcomes with four knots defined at the fifth, 35, 65, and 95th percentiles of HALP score. Statistical analysis was performed using IBM SPSS software version 24.0 (SPSS, Inc., Chicago, IL, USA) and R (version 3.5.0). A two-tailed value of *P* < 0.05 was considered statistically significant.

## Results

### Baseline Characteristics

**Figure 1:**
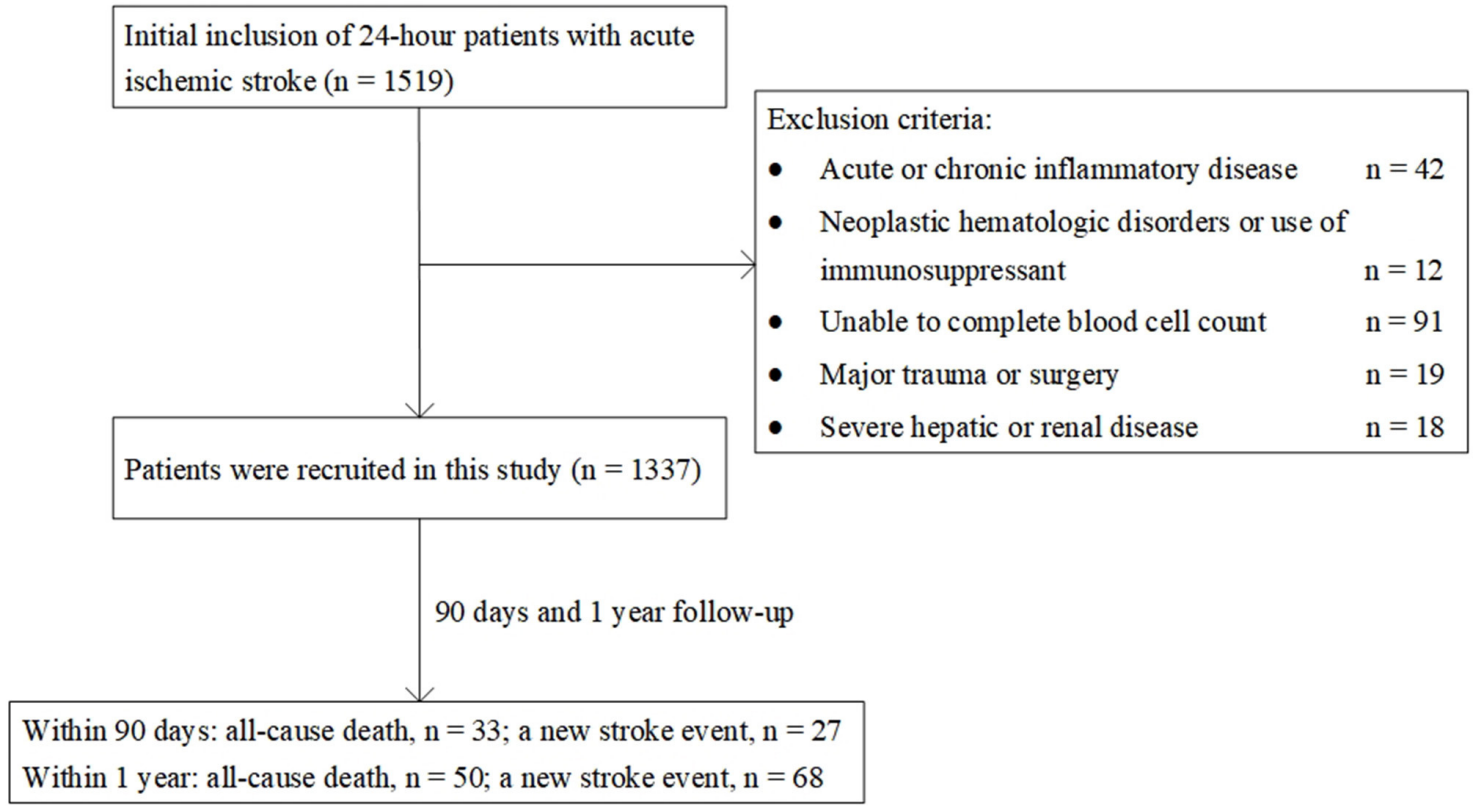
Patient flowchart of the cohort.

A total of 1,337 patients were included in our analysis (Figure 1). The mean age of the patients was 60.55 years, and 30.5% of them were female. The study population was divided into three tertiles based on the HALP score value (Table 1). Compared with the higher HALP score patients, those with a lower HALP score were more likely to be older; be female; have atrial fibrillation; have lower lymphocyte, hemoglobin, and albumin levels; and have higher platelet counts, frequencies of cardioembolic stroke, and baseline NIHSS scores.

**Table 1:** Baseline characteristics of patients according to the HALP score tertiles.

		**HALP score tertiles**			
**Variable**	**Overall**	**Tertile 1 <36.65**	**Tertile 2 36.65–54.42**	**Tertile 3 >54.42**	***P* for trend**
Patients, *n*	1,337	445	446	446	–
Age, years, mean ± SD	60.55 ± 12.45	63.20 ± 12.45	60.24 ± 11.92	58.20 ± 12.48	<0.001
Female, *n* (%)	408 (30.5)	179 (40.2)	123 (27.6)	106 (23.8)	<0.001
Smoking, *n* (%)	585 (43.8)	198 (44.5)	190 (42.6)	197 (44.2)	0.923
Time from onset to blood sampling, h, median (IQR)	17 (13-21)	16 (11-21)	17 (14-22)	17 (14-22)	0.345
**Medical history**, ***n*** **(%)**					
Hypertension	809 (60.5)	274 (61.6)	268 (60.1)	267 (59.9)	0.602
Diabetes	311 (23.3)	97 (21.8)	97 (21.7)	117 (26.2)	0.117
Stroke history	322 (24.1)	114 (25.6)	99 (22.2)	109 (24.4)	0.681
Coronary heart disease	159 (11.9)	61 (13.7)	48 (10.8)	50 (11.2)	0.250
Atrial fibrillation	125 (9.3)	64 (14.4)	36 (8.1)	25 (5.6)	<0.001
IV thrombolysis treatment, n (%)	294 (22.0)	114 (25.6)	91 (20.4)	89 (20.0)	0.041
Baseline NIHSS score, median (IQR)	3 (2-6)	4 (2-8)	3 (2-6)	3 (1-5)	<0.001
Hemoglobin, g/l, median (IQR)	138 (128–148)	132 (120–143)	138 (130–147)	144 (134–153)	<0.001
Albumin, g/l, median (IQR)	40.9 (38.5–43.1)	39.6 (37.4–42.1)	40.9 (38.9–43.1)	41.8 (39.6–44.1)	<0.001
Lymphocyte, 10^9^/l, median (IQR)	1.7 (1.3–2.2)	1.2 (0.94–1.5)	1.7 (1.4–2.0)	2.2 (1.9–2.6)	<0.001
Platelet, 10^9^/l, median (IQR)	207 (173–250)	234 (192–279)	209 (176–247)	186 (159–218)	<0.001
**Stroke etiology**, ***n*** **(%)**					
Large artery atherosclerosis	511 (38.2)	164 (36.9)	170 (38.1)	177 (39.7)	0.385
Small artery disease	329 (24.6)	100 (22.5)	119 (26.7)	110 (24.7)	0.448
Cardioembolic stroke	173 (13.0)	80 (18.0)	51 (11.4)	43 (9.6)	<0.001
Stroke of other determined etiology	46 (3.4)	18 (4.0)	16 (3.6)	12 (2.7)	0.268
Stroke of undetermined etiology	277 (20.7)	83 (18.7)	90 (20.4)	104 (23.3)	0.086

*HALP, hemoglobin, albumin, lymphocyte, and platelet; NIHSS, National Institutes of Health Stroke Scale*.

### Association Between HALP Score and Poor Outcome

**Figure 2:**
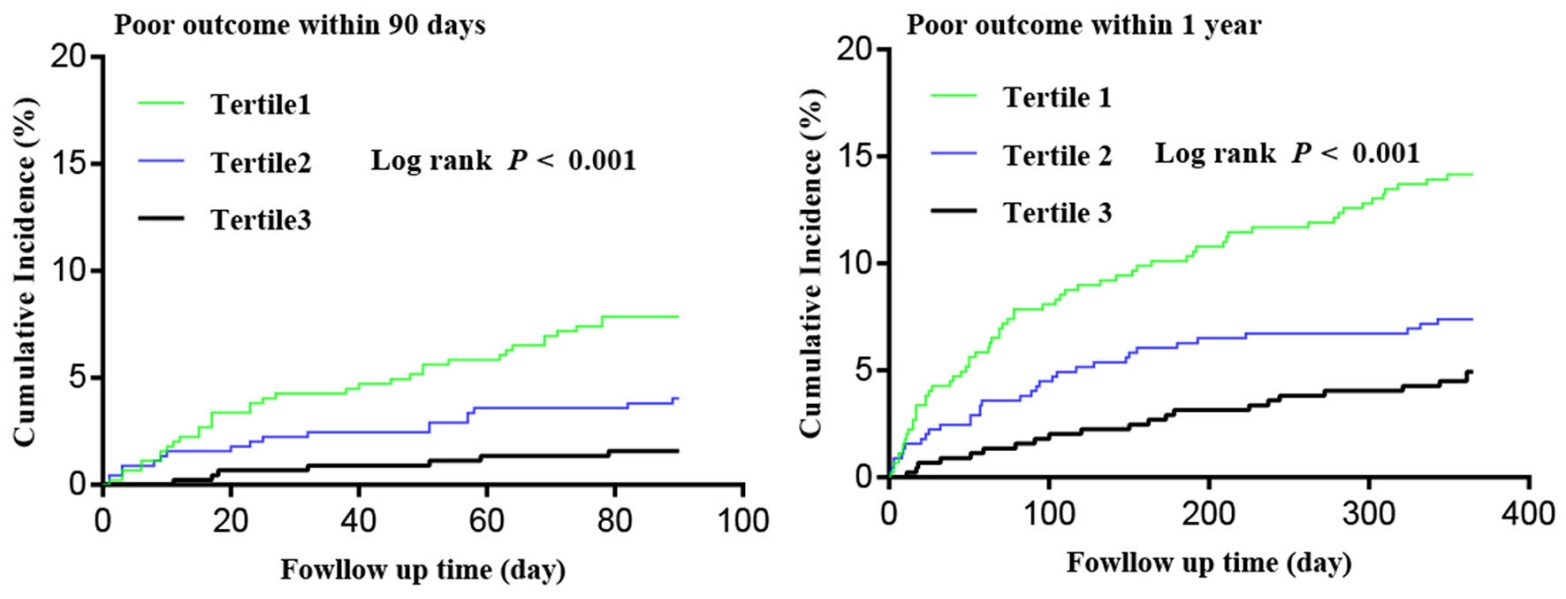
Kaplan–Meier curves of cumulative incidence (%) of poor outcome by tertiles of hemoglobin, albumin, lymphocyte, and platelet (HALP) score at 90-day and 1-year follow-up.

After 90 days of follow-up, 60 (4.5%) patients were lost to follow-up and 60 (4.5%) patients experienced poor outcome. At the 1-year follow-up, 92 (6.9%) patients were lost and 118 (8.8%) patients experienced poor outcome (Figure 1). All Kaplan–Meier curves showed that patients in the lowest tertile of HALP score (<36.65) had the highest cumulative incidence of poor outcome within 90 days and at 1 year (log-rank *P* < 0.05 for all; Figure 2).

After adjustment for age, sex, history of hypertension, diabetes, ischemic stroke, coronary heart disease, atrial fibrillation, smoking, time from onset to blood sampling, IV thrombolysis treatment and baseline NIHSS score, higher levels of HALP score were associated with decreased risk of poor outcome within 90 days and 1 year (Table 2). The adjusted HRs (95% confidence intervals) for the highest vs. lowest tertile of HALP score were 0.25 (0.11–0.57) for poor outcome at 90 days and 0.42 (0.25–0.69) at 1 year. Similarly, on continuous analyses, each 1-SD increase of the HALP score was associated with 90-day and 1-year poor outcome (Table 2). ROC curve analysis of HALP score and poor outcome at 90-day and 1-year follow-up indicated that optimal cutoff values for HALP score were 33.82 and 32.77, respectively. The sensitivity and specificity were 53.3 and 75.9% (C statistic: 0.69, 95% CI: 0.66–0.71, *P* < 0.001) for 90-day poor outcome, and for 1-year poor outcome, they were 49.2 and 74.3% (C statistic: 0.65, 95% CI: 0.62–0.67, *P* < 0.001).

**Table 2:** HRs (95% CIs) for poor outcome according to HALP score tertiles.

	**Poor outcome within 90 days**			**Poor outcome within 1 year**		
**HALP score**	**Events, *n* (%)**	**Unadjusted Model**	Adjusted Model^*^	**Events, *n* (%)**	**Unadjusted Model**	Adjusted Model^*^
Tertile 1	35 (7.9)	Reference	Reference	63 (14.2)	Reference	Reference
Tertile 2	18 (4.0)	0.50 (0.29–0.89)	0.61 (0.34–1.10)	33 (7.4)	0.51 (0.33–0.77)	0.60 (0.39–0.92)
Tertile 3	7 (1.6)	0.19 (0.09–0.44)	0.25 (0.11–0.57)	22 (4.9)	0.33 (0.20–0.54)	0.42 (0.25–0.69)
*P* for trend	<0.001	<0.001	0.001	<0.001	<0.001	<0.001
Per SD increment	60 (4.5)	0.96 (0.95–0.98)	0.97 (0.96–0.99)	118 (8.8)	0.98 (0.97–0.99)	0.98 (0.97–0.99)

*HR, hazard ratio; CI, confidence interval; SD, standard deviation; HALP, hemoglobin, albumin, lymphocyte, and platelet; NIHSS, National Institutes of Health Stroke Scale*.

* *Adjusted for age, sex, history of hypertension, diabetes, ischemic stroke, coronary heart disease and atrial fibrillation, smoking, time from onset to blood sampling, intravenous (IV) thrombolysis treatment, and baseline NIHSS scores*.

**Figure 3:**
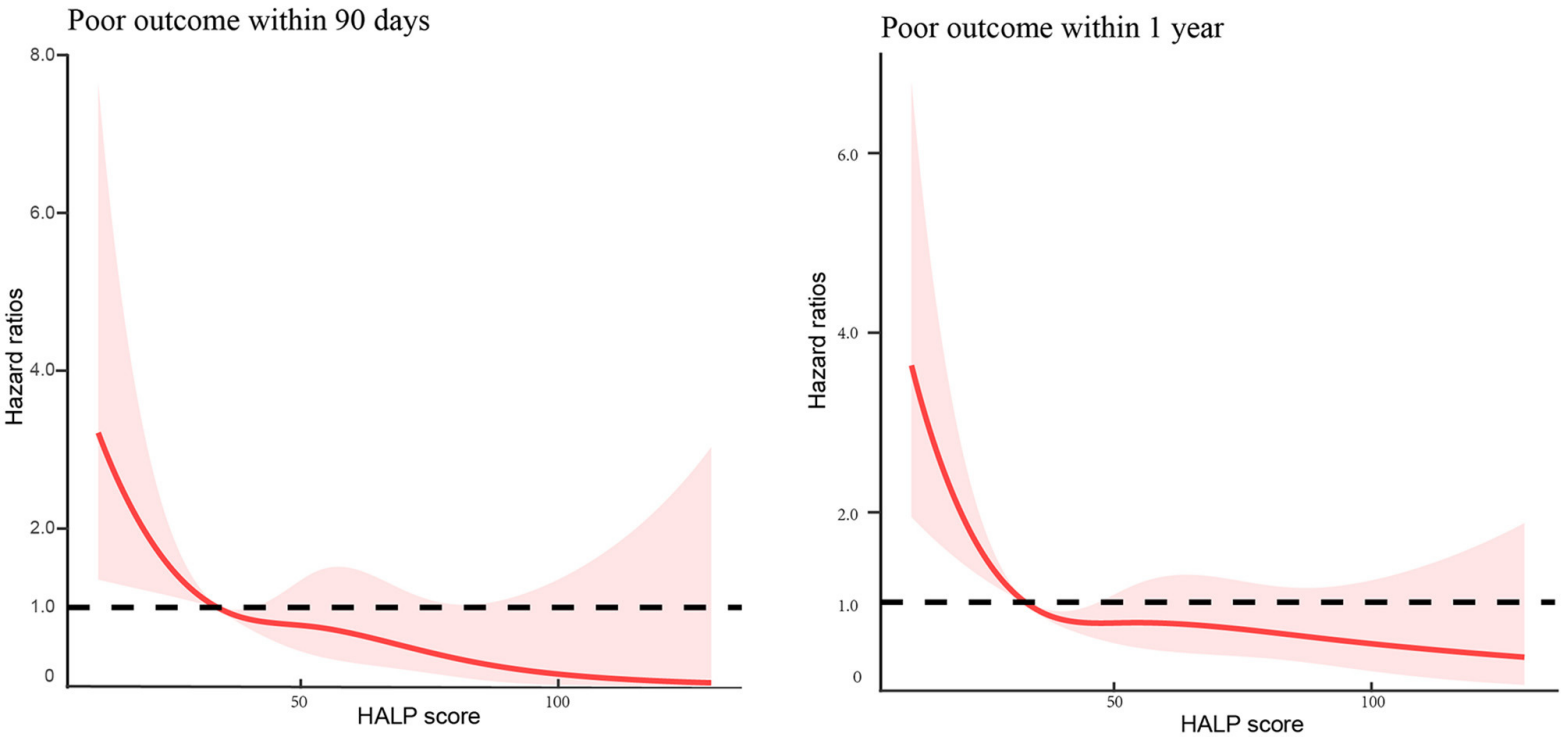
Association of hemoglobin, albumin, lymphocyte, and platelet (HALP) score with the risk of poor outcome among patients with acute ischemic stroke at 90-day and 1-year follow-up.

In addition, multivariable-adjusted spline regression models showed J-shaped associations between HALP score and the risk of poor outcome within 90 days and 1 year (Figure 3).

### Incremental Predictive Ability of HALP Score

Adding HALP score to a multivariable model, which included age, sex, history of hypertension, diabetes, ischemic stroke, coronary heart disease, atrial fibrillation, smoking, intravenous (IV) thrombolysis treatment, and baseline NIHSS scores, significantly improved its predictive ability (Table 3). The continuous net reclassification improvement was 48.38% (*P* < 0.001) and 28.95% (*P* = 0.002) for 90-day and 1-year poor outcome, respectively. IDI was 1.51% (*P* = 0.027) and 1.51% (*P* < 0.001) for 90-day and 1-year poor outcome, respectively.

**Table 3:** Reclassification and discrimination statistics for poor outcome by HALP score.

	**C Statistic**		**NRI (Continuous), %**		**IDI, %**	
	**Estimate (95% CI)**	***P*-value**	**Estimate (95% CI)**	***P*-value**	**Estimate (95% CI)**	***P-*value**
**Poor outcome within 90 days**						
Model 1	0.71 (0.69–0.74)		Reference		Reference	
Model 1 + HALP score	0.74 (0.72–0.77)	0.146	48.38 (24.55–72.21)	<0.001	1.51 (0.18–2.84)	0.027
**Poor outcome within 1 year**						
Model 1	0.64 (0.62–0.67)		Reference		Reference	
Model 1 + HALP score	0.68 (0.65–0.71)	0.070	28.95 (10.87–47.03)	0.002	1.51 (0.74–2.29)	<0.001

*Model 1 adjusted for age, sex, history of hypertension, diabetes, ischemic stroke, coronary heart disease and atrial fibrillation, smoking, intravenous (IV) thrombolysis treatment, and baseline NIHSS scores. HALP, hemoglobin, albumin, lymphocyte, and platelet; CI, confidence interval; IDI, integrated discrimination improvement; NIHSS, National Institutes of Health Stroke Scale; NRI, net reclassification improvement*.

## Discussion

We explored the prognostic value of the novel index HALP score in patients with AIS in this prospective cohort study and found that higher levels of HALP score at admission were highly associated with a decreased risk of recurrent stroke and death within 90 days and 1 year. Furthermore, we demonstrated that the addition of HALP score to the conventional risk factors could increase the discriminatory power and risk reclassification for recurrent stroke and death. These results suggested that decreased HALP score could be an independent risk factor of recurrent stroke and death.

Ischemic stroke initiates with gradual or sudden cerebral hypoperfusion, including oxidative stress, hemostatic activation, and inflammation, and eventually leads to a corresponding loss of neurological function (17). Ischemic brain tissue activates leukocytes and promotes their migration to the ischemic site by releasing pro-inflammatory chemokines (18). The inflammation triggers the process of thrombosis, in which platelets participate in adhesion, release reaction, and aggregation (6). Studies have shown that platelet count may be a qualified predictor for long-term recurrent stroke, mortality, and poor functional outcome (19). Lymphocytes play an important role in the elimination and repair of inflammation; lower lymphocyte counts were associated with increased infarct volume, neurological deterioration, and poor prognosis after ischemic stroke (20). Albumin has a neuroprotective effect because of its antagonism of oxidation, stagnant, thrombosis, and leukocyte adhesion (21, 22). Serum albumin levels can predict the prognosis of ischemic stroke (23). Hemoglobin has oxygen-carrying capacity and can affect the energy balance in the penumbra (24). There is a U-shaped association between hemoglobin and poor prognosis of ischemic stroke (25). Studies have shown that a high hemoglobin concentration is associated with carotid atherosclerosis and may be a risk factor for ischemic stroke (26). Similarly, low hemoglobin and hematocrit levels are strongly associated with poor outcome and mortality after AIS (27). Further, serum albumin and hemoglobin concentrations have a predictive value for stroke recurrence and combined events (28, 29). The new indicator HALP score is based on the combination of the four hematological parameters mentioned above.

Recent studies have shown that the HALP score can reflect the inflammation–nutritional status of patients (9, 30) and has been proved to be an important prognostic indicator for patients with multiple tumors (10–12). Anemia and thrombosis could exacerbate inflammation, while lymphocytes reduce inflammation (26). Serum albumin has been considered an indicator of nutritional status. Some studies have also suggested that albumin reflects the severity of inflammation and illness in acute diseases (31). It is widely accepted that the inflammatory response and nutritional status are correlated with the prognosis of patients with stroke (4, 32, 33). HALP score is obtained by hemoglobin (g/L) × albumin (g/L) × lymphocytes (/L)/platelets (/L), which makes it a cost-effective, simple parameter to easily assess the inflammation–nutritional status. This finding may be significant because instant inflammation–nutritional status assessment can help clinicians assess prognosis and formulate appropriate treatment plans.

There were some limitations to this study. First, this was a single-center prospective study. Although we included patients in three branches, there was still a possibility of selection bias. Second, the potential influence of concomitant medications, such as thrombolytic therapy, oral anti-platelet, and statins, were not considered. Third, we only obtained the hemoglobin count, albumin levels, lymphocyte count, and platelet count at admission but did not present the dynamic change of HALP score at different stages. Therefore, multicenter cohort studies are still needed to validate the findings.

## Conclusion

Our study indicated an association between HALP score and the risk of recurrent stroke and death within 90 days and 1 year, suggesting that HALP score at admission may act as a powerful indicator of recurrent stroke and death in patients with AIS.

## Data Availability Statement

The raw data supporting the conclusions of this article will be made available by the authors, without undue reservation.

## Ethics Statement

The studies involving human participants were reviewed and approved by the ethics committee of the First Affiliated Hospital of Zhengzhou University. The patients/participants provided their written informed consent to participate in this study.

## Author Contributions

BS and YX designed the overall study with contributions from MN, FB, JW, and SS. MT designed and carried out experiments and collected data with YL, XiaW, XT, L-lP, XinW, LZ, and WS. MT wrote the manuscript. BS supervised this study, designed experiments, and edited the paper. All authors critically reviewed the manuscript and approved the submitted version.

## Conflict of Interest

The authors declare that the research was conducted in the absence of any commercial or financial relationships that could be construed as a potential conflict of interest.

## References

[1] Mozaffarian D Benjamin EJ Go AS Arnett DK Blaha MJ Cushman M Heart disease and stroke statistics-2016 update: a report from the American Heart Association. Circulation. (2016) 133:e38-360. 10.1161/CIR.0000000000000350 26673558

[2] Esenwa CC Elkind MS. Inflammatory risk factors, biomarkers and associated therapy in ischaemic stroke. Nat Rev Neurol. (2016) 12:594–604. 10.1038/nrneurol.2016.125 27615422

[3] de Lau LM Leebeek FW de Maat MP Koudstaal PJ Dippel DW. A review of hereditary and acquired coagulation disorders in the aetiology of ischaemic stroke. Int J Stroke. (2010) 5:385–94. 10.1111/j.1747-4949.2010.00468.x 20854623

[4] Collaboration. FT. Poor nutritional status on admission predicts poor outcomes after stroke: observational data from the FOOD trial. Stroke. (2003) 34:1450–6. 10.1161/01.STR.0000074037.49197.8C 12750536

[5] Baird AE. The forgotten lymphocyte: immunity and stroke. Circulation. (2006) 113:2035–6. 10.1161/CIRCULATIONAHA.105.620732 16651483

[6] Reininger AJ Bernlochner I Penz SM Ravanat C Smethurst P Farndale RW. A 2-step mechanism of arterial thrombus formation induced by human atherosclerotic plaques. J Am Coll Cardiol. (2010) 55:1147–58. 10.1016/j.jacc.2009.11.051 20223370

[7] Milionis H Papavasileiou V Eskandari A D'Ambrogio-Remillard S Ntaios G Michel P Anemia on admission predicts short- and long-term outcomes in patients with acute ischemic stroke. Int J Stroke. (2015) 10:224–30. 10.1111/ijs.12397 25427291

[8] Tanne D Molshatzki N Merzeliak O Tsabari R Toashi M Schwammenthal Y. Anemia status, hemoglobin concentration and outcome after acute stroke: a cohort study. BMC Neurol. (2010) 10:22. 10.1186/1471-2377-10-22 20380729 PMC2858127

[9] Xu SS Li S Xu HX Li H Wu CT Wang WQ. Haemoglobin, albumin, lymphocyte and platelet predicts postoperative survival in pancreatic cancer. World J Gastroenterol. (2020) 26:828–38. 10.3748/wjg.v26.i8.828 32148380 PMC7052532

[10] Shen XB Zhang YX Wang W Pan YY. The hemoglobin, albumin, lymphocyte, and platelet (HALP) score in patients with small cell lung cancer before first-line treatment with etoposide and progression-free survival. Med Sci Monit. (2019) 25:5630–9. 10.12659/MSM.917968 31356586 PMC6685331

[11] Cong L Hu L. The value of the combination of hemoglobin, albumin, lymphocyte and platelet in predicting platinum-based chemoradiotherapy response in male patients with esophageal squamous cell carcinoma. Int Immunopharmacol. (2017) 46:75–9. 10.1016/j.intimp.2017.02.027 28268208

[12] Peng D Zhang CJ Gong YQ Hao H Guan B Li XS. Prognostic significance of HALP (hemoglobin, albumin, lymphocyte and platelet) in patients with bladder cancer after radical cystectomy. Sci Rep. (2018) 8:794. 10.1038/s41598-018-19146-y 29335609 PMC5768698

[13] Guo Y Shi D Zhang J Mao S Wang L Zhang W. The hemoglobin, albumin, lymphocyte, and platelet (HALP) score is a novel significant prognostic factor for patients with metastatic prostate cancer undergoing cytoreductive radical prostatectomy. J Cancer. (2019) 10:81–91. 10.7150/jca.27210 30662528 PMC6329846

[14] Song B Hu R Pei L Cao Y Chen P Sun S. Dual antiplatelet therapy reduced stroke risk in high-risk patients with transient ischaemic attack assessed by ABCD3-I score. Eur J Neurol. (2019) 26:610–6. 10.1111/ene.13864 30414298

[15] Zhao L Wang R Song B Tan S Gao Y Fang H. Association between atherogenic dyslipidemia and recurrent stroke risk in patients with different subtypes of ischemic stroke. Int J Stroke. (2015) 10:752–8. 10.1111/ijs.12471 25924059

[16] Stroke−1989. Recommendations on stroke prevention, diagnosis, and therapy. Report of the WHO Task Force on Stroke and other Cerebrovascular Disorders. Stroke. (1989) 20:1407–31. 10.1161/01.STR.20.10.1407 2799873

[17] Brouns R De Deyn PP. The complexity of neurobiological processes in acute ischemic stroke. Clin Neurol Neurosurg. (2009) 111:483–95. 10.1016/j.clineuro.2009.04.001 19446389

[18] Kim JY Park J Chang JY Kim SH Lee JE. Inflammation after ischemic stroke: the role of leukocytes and glial cells. Exp Neurobiol. (2016) 25:241–51. 10.5607/en.2016.25.5.241 27790058 PMC5081470

[19] Yang M Pan Y Li Z Yan H Zhao X Liu L. Platelet count predicts adverse clinical outcomes after ischemic stroke or TIA: subgroup analysis of CNSR II. Front Neurol. (2019) 10:370. 10.3389/fneur.2019.00370 31031698 PMC6473473

[20] Kim J Song TJ Park JH Lee HS Nam CM Nam HS. Different prognostic value of white blood cell subtypes in patients with acute cerebral infarction. Atherosclerosis. (2012) 222:464–7. 10.1016/j.atherosclerosis.2012.02.042 22460048

[21] Taverna M Marie AL Mira JP Guidet B. Specific antioxidant properties of human serum albumin. Ann Intensive Care. (2013) 3:4. 10.1186/2110-5820-3-4 23414610 PMC3577569

[22] Lam FW Cruz MA Leung HC Parikh KS Smith CW Rumbaut RE. Histone induced platelet aggregation is inhibited by normal albumin. Thromb Res. (2013) 132:69–76. 10.1016/j.thromres.2013.04.018 23673386

[23] Dziedzic T Slowik A Szczudlik A. Serum albumin level as a predictor of ischemic stroke outcome. Stroke. (2004) 35:e156–8. 10.1161/01.STR.0000126609.18735.be 15073386

[24] Kimberly WT Lima FO O'Connor S Furie KL. Sex differences and hemoglobin levels in relation to stroke outcomes. Neurology. (2013) 80:719–24. 10.1212/WNL.0b013e31828250ff 23365064 PMC3589294

[25] Sacco S Marini C Olivieri L Pistoia F Carolei A. Contribution of hematocrit to early mortality after ischemic stroke. European neurology. (2007) 58:233–8. 10.1159/000107946 17827968

[26] Barlas RS Honney K Loke YK McCall SJ Bettencourt-Silva JH Clark AB. Impact of hemoglobin levels and anemia on mortality in acute stroke: analysis of UK Regional Registry Data, Systematic Review, and Meta-Analysis. J Am Heart Assoc. (2016) 5:e003019. 10.1161/JAHA.115.003019 27534421 PMC5015269

[27] Kellert L Martin E Sykora M Bauer H Gussmann P Diedler J. Cerebral oxygen transport failure?: decreasing hemoglobin and hematocrit levels after ischemic stroke predict poor outcome and mortality: STroke: RelevAnt Impact of hemoGlobin, Hematocrit and Transfusion (STRAIGHT)–an observational study. Stroke. (2011) 42:2832–7. 10.1161/STROKEAHA.110.606665 21852622

[28] Zhang Q Lei YX Wang Q Jin YP Fu RL Geng HH. Serum albumin level is associated with the recurrence of acute ischemic stroke. Am J Emerg Med. (2016) 34:1812–6. 10.1016/j.ajem.2016.06.049 27364646

[29] Chang JY Lee JS Kim BJ Kim JT Lee J Cha JK Influence of hemoglobin concentration on stroke recurrence and composite vascular events. Stroke. (2020) 51:1309–12. 10.1161/STROKEAHA.119.028058 32078481

[30] Peng D Zhang CJ Tang Q Zhang L Yang KW Yu XT. Prognostic significance of the combination of preoperative hemoglobin and albumin levels and lymphocyte and platelet counts (HALP) in patients with renal cell carcinoma after nephrectomy. BMC Urol. (2018) 18:20. 10.1186/s12894-018-0333-8 29544476 PMC5855974

[31] Eckart A Struja T Kutz A Baumgartner A Baumgartner T Zurfluh S. Relationship of nutritional status, inflammation, and serum albumin levels during acute illness: a prospective study. Am J Med. (2019) 133:713–22.e7. 10.1016/j.amjmed.2019.10.031 31751531

[32] Fu Y Liu Q Anrather J Shi FD. Immune interventions in stroke. Nat Rev Neurol. (2015) 11:524–35. 10.1038/nrneurol.2015.144 26303850 PMC4851339

[33] Martineau J Bauer JD Isenring E Cohen S. Malnutrition determined by the patient-generated subjective global assessment is associated with poor outcomes in acute stroke patients. Clin Nutr. (2005) 24:1073–7. 10.1016/j.clnu.2005.08.010 16213064

[34] Mengke T Youfeng L Xiao W Xuan T Lu-lu P Xin W Hemoglobin, albumin, lymphocyte, and platelet score is associated with adverse clinical outcomes of acute ischemic stroke: a prospective cohort study. Res Square. (2020). 10.21203/rs.3.rs-38304/v1

